# A Global Survey of Emergency Department Responses to the COVID-19 Pandemic

**DOI:** 10.5811/westjem.2021.3.50358

**Published:** 2021-08-21

**Authors:** Prashant Mahajan, Chong Shu-Ling, Camilo Gutierrez, Emily White, Benjamin A.Y. Cher, Elizabeth Freiheit, Apoorva Belle, EMERGE NETWORK, Johanna Kaartinen, Vijaya Arun Kumar, Paul M. Middleton, Chip Jin Ng, Daniel Osei-Kwame, Dominik Roth, Tej Prakash Sinja, Sagar Galwankar, Michele Nypaver, Nathan Kuppermann, Ulf EKelund

**Affiliations:** * University of Michigan, Departments of Emergency Medicine and Pediatrics, Ann Arbor, Michigan; † KK Women’s and Children’s Hospital, Department of Emergency Medicine, Singapore; ‡ George Washington University School of Medicine and Health Sciences, Departments of Emergency Medicine and Pediatrics, Washington, District of Columbia; § University of Michigan, SABER, Ann Arbor, Michigan; ¶ University of Michigan Medical School, Ann Arbor, Michigan; || University of Michigan EMERGE, Department of Emergency Medicine, Ann Arbor, Michigan; # See supplemental file for full authorship; * University of Helsinki/Helsinki University Hospital, Department of Emergency Medicine and Services, Helsinki, Finland; † Wayne State University, Department of Emergency Medicine, Detroit, Michigan; ‡ South Western Emergency Research Institute, Department of Emergency Medicine, Liverpool, England; § Chang Gung Memorial Hospital, Department of Emergency Medicine, Taoyuan City, Taiwan; ¶ Komfo Anokye Teaching Hospital, Department of Emergency Medicine, Kumasi, Ghana; || Medical University of Vienna, Department of Emergency Medicine, Vienna, Austria; # All India Institute of Medical Sciences, Department of Emergency Medicine, New Delhi, India; ** Sarasota Memorial Hospital, Department of Emergency Medicine, Sarasota, Florida; †† University of Michigan, Department of Emergency Medicine, Ann Arbor, Michigan; ‡‡ University of California, Davis School of Medicine, Departments of Emergency Medicine and Pediatrics, Davis, California; §§ Skane University at Lund, Department of Emergency Medicine, Lund, Sweden

## Abstract

**Introduction:**

Emergency departments (ED) globally are addressing the coronavirus disease 2019 (COVID-19) pandemic with varying degrees of success. We leveraged the 17-country, Emergency Medicine Education & Research by Global Experts (EMERGE) network and non-EMERGE ED contacts to understand ED emergency preparedness and practices globally when combating the COVID-19 pandemic.

**Methods:**

We electronically surveyed EMERGE and non-EMERGE EDs from April 3–June 1, 2020 on ED capacity, pandemic preparedness plans, triage methods, staffing, supplies, and communication practices. The survey was available in English, Mandarin Chinese, and Spanish to optimize participation. We analyzed survey responses using descriptive statistics.

**Results:**

74/129 (57%) EDs from 28 countries in all six World Health Organization global regions responded. Most EDs were in Asia (49%), followed by North America (28%), and Europe (14%). Nearly all EDs (97%) developed and implemented protocols for screening, testing, and treating patients with suspected COVID-19 infections. Sixty percent responded that provider staffing/back-up plans were ineffective. Many sites (47/74, 64%) reported staff missing work due to possible illness with the highest provider proportion of COVID-19 exposures and infections among nurses.

**Conclusion:**

Despite having disaster plans in place, ED pandemic preparedness and response continue to be a challenge. Global emergency research networks are vital for generating and disseminating large-scale event data, which is particularly important during a pandemic.

## INTRODUCTION

Emergency departments (ED) globally are on the front lines in addressing the COVID-19 pandemic, caused by the severe acute respiratory syndrome coronavirus 2 (SARS-CoV-2). Most EDs have disaster preparedness plans in place for health system responses to large-scale disasters, including infectious disease outbreaks. However, infectious disease pandemics pose a unique challenge due to their infrequency and the extended period over which they may occur.

The incomplete and evolving knowledge of a novel pathogen limits early preparations for resource needs during a pandemic and predisposes individuals and communities to poor health outcomes. Critical evaluation of the global response to the 2009 H1N1 influenza pandemic identified substantial variability and poorly conceived or even absent preparedness plans in many emergency care systems.^1^ This missed opportunity to implement successful disaster response plans prior to the following major infectious outbreak highlights the need to study global ED responses and healthcare system preparedness on a continuous basis.^2^,^3^ With the ongoing COVID-19 pandemic, knowledge regarding presentation, prognosis, and response to therapies continues to evolve. It is imperative for data, lessons learned, and successful approaches used by EDs with a high pandemic burden to be made rapidly and reliably available to those in earlier stages.

Emergency medicine networks are valuable for collection of data supporting research, administrative, and educational goals and can potentially be leveraged to collate and disseminate experiences from disasters.^4^ Emergency Medicine Education & Research by Global Experts (EMERGE) is a newly developed network of 26 EDs across 17 countries and six continents whose goal is to improve the care of acutely ill and injured patients by garnering the collective experiences of its member EDs.^5^,^6^

In this study we sought to leverage the EMERGE network and establish collaborations with non-EMERGE EDs to determine global ED preparedness for COVID-19 and, specifically, to identify successful processes and protocols that may be adopted and/or adapted by other EDs to improve patient outcomes.

## METHODS

### Study Design and Survey Development

We performed a cross-sectional study of ED practices during the pandemic using a survey sent to all participating sites. We reported our results using elements from the Strengthening the Reporting of Observational Studies in Epidemiology; Survey Reporting Guidelines; and the Checklist for Reporting Results of Internet E-Surveys guidelines.^7^–^10^ We used the online survey platform Qualtrics (Qualtrics LLC, Provo, Utah) to characterize ED COVID-19 responses between April 3–June 1, 2020 along the following domains: pandemic preparedness plans and training; physical space; triage methods unique to the pandemic; staffing; supplies; and communication practices. The survey (Appendix A) was piloted and assessed for response process validity. Eleven emergency physicians at the University of Michigan evaluated the survey for construct and face-validity, and iteratively refined the questions. Survey questions were mainly closed-ended, with additional open-ended questions allowing participants to express opinions or to provide clarification on responses. The survey was translated using formal translation services into Mandarin Chinese and Spanish and reassessed for face validity in the translated language by EMERGE Executive Committee members who spoke those languages. The study was determined not to require regulation as human subjects research by the University of Michigan Institutional Review Board (HUM00178847).

**Population Health Research Capsule**

What do we already know about this issue?

*Emergency departments (EDs) globally are on the front lines in addressing the coronavirus disease 2019 pandemic. However, preparedness and effectiveness of responses remain unknown*.

What was the research question?

*We leveraged the 17-country, Emergency Medicine Education and Research by Global Experts (EMERGE) network and non-EMERGE EDs to study emergency preparedness globally*.

What was the major finding of the study?

*EDs had to rapidly update and modify preparedness plans. EDs developed innovative processes to respond, and most identified provider burnout as an important issue*.

How does this improve population health?

*Emergency research networks are vital for generating and disseminating solutions to improve patient outcomes in global emergencies*.

### Participants and Survey Distribution

Survey participation was voluntary, and respondents did not receive financial remuneration. The survey was distributed to EMERGE member institutions via email with unique links created for each participant. Attempts were made to involve non-EMERGE institutions by forwarding a one-page infographic (Appendix B) to contacts of EMERGE members and associates. Simultaneously, we contacted other international emergency associations for participation, including the Pan-Asian Resuscitation Outcomes Study, the World Academic Congress of Academic Medicine, the Michigan Emergency Department Improvement Collaborative, and the International Federation for Emergency Medicine.^11^–^14^ To encourage survey completion, we stated the deadline for survey responses and sent regular email reminders about survey closure. We also contacted participants electronically to obtain clarification on incomplete or partially complete responses. We specified that the medical director or emergency preparedness expert in the division/department should be the one to answer this questionnaire.

### Statistical Analysis

We tabulated descriptive statistics including absolute and relative frequencies for categorical variables and means (with standard deviations [SD]) or medians (with interquartile ranges [IQR]) for continuous variables, depending on normality, to compare all survey question responses. We defined ED characteristics by location, setting, and size. We grouped EDs by location into geographical categories: Asia; Europe; North America; South America; Africa; and Australia. Hospital setting was defined along two domains. First, we defined them as “public” or “government-funded” hospitals with the rest defined as “private” hospitals. Second, we categorized hospitals as “academic” vs “non-academic” based on presence of residency training programs. We used SAS 9.4 (SAS Institute, Cary, NC) for all quantitative analyses. ED characteristics stratified by country, continent, and income status (gross domestic product) as well as COVID-19 prevalence and death rates at time of survey completion are provided in Supplementary Table 1.

## RESULTS

### Respondent Characteristics

We identified 129 EDs across 28 countries within all six World Health Organization (WHO) regions. Of these, 74 (57%) completed the survey, comprising 23/26 (88%) EMERGE EDs and 51/103 (49%) non-EMERGE EDs (Figure). There were 21 (28%) respondents from North America, 36 (49%) from Asia, 10 (14%) from Europe, two (3%) from South America, two (3%) from Africa, and three (4%) from Australia. With 69 sites providing their daily volumes, we approximated the total annual patient population of the represented EDs at 6,068,994. The median ED bed count was 42 (IQR 21–80) and median ED encounters daily was 180 (IQR 100 – 300); 52/74 sites are both pediatric and adult EDs, 14 are adult only, and eight are pediatric only.

### Pandemic Preparedness

Most sites (71/74, 96%) reported having an ED protocol to guide the screening, testing, and managing of suspected COVID-19 cases, and 27 (36%) sites sent their protocols for other EDs across the globe to use. There were 43/74 EDs (58%) that had ED staffing back-up plans prior to the COVID-19 outbreak. Regarding supply availability, 59/71 (83%) reported stocking the ED with personal protective equipment (PPE) as a part of pre-existing disaster plans. However, when respondents answered questions regarding effectiveness of existing disaster plans only 59/74 (80%) replied. Of these, 48/59 (81%) responded that the plan was successful/effective. Regarding personnel and ED staffing back-up plans, 26 felt they were very effective, 33 somewhat effective, six not effective, and nine did not respond. Reasons for disaster plans being successful or not are described in Table 1.

### COVID-19 Pandemic Response

Nearly every ED (71/73, 97%) developed and implemented a protocol for screening, testing, and managing patients with suspected SARS-CoV-2 infections. The WHO recommendations for COVID-19 informed the pandemic response plans for 28/73 (38%) EDs in our sample. Outside the United States, 13/59 (22%) EDs based their pandemic response plans on the US Centers for Disease Control and Prevention (CDC) guidelines. The majority (45/73, 62%) based their plans on guidelines issued by their own countries.

### ED Triage and Capacity

Screening criteria for COVID-19 were generally similar across the EDs in our survey, with less similarity across survey respondents in approaches for increasing treatment capacity. Screening criteria used are provided in Table 2 with the most common being fever (72/74, 97%), close contact with a confirmed case of COVID-19 (66/74, 89%), travel to a COVID-19 affected area (66/74, 89%), symptoms of upper respiratory illness (63/74, 85%), and signs of lower respiratory illness (71/74, 96%). However, 71/74 (96%) EDs reported that triage screening criteria had changed over time, and 58/74 (78%) mentioned criteria had changed more than two times. Of those responding, 68/74 (92%) created separate COVID-19 areas in ED waiting rooms, and 51/72 EDs (71%) increased capacity in response to COVID-19 surges, with 46 enhancing existing ED space by modifying/adding hallway beds, chairs/recliners or creating spaces separated by curtains. The results show that 45/74 (61%) increased capacity outside the ED by using non-traditional space for ED care such as subspecialty clinics or mobile tents. The most common measures used to increase hospital capacity were postponing elective/non-urgent surgical procedures (67/74, 91%), creating a dedicated COVID-19 patient care team (57/74, 77%), and reassigning existing beds specifically for patients with COVID-19 (56/74, 76%).

### ED Staffing and Staff Wellness/Burnout

Many survey respondents identified staffing issues as a particularly challenging aspect of the COVID-19 pandemic response. Most respondents (52/74, 70%) developed new or separate staff backup plans specifically for COVID-19 while 22/74 EDs (30%) had activated their existing staff backup plans at the time the survey was completed. From the received responses 47/74 (64%) reported staff missing work due to illness, and 32/74 (43%) reported that ED provider staff had tested positive for SARS-CoV-2. Inability to work due to possible COVID-19 illness was reported in nurses in 26/32 (81%) responses, followed by physicians, 21/32 (66%), and residents, 20/32 (63%). Common measures to address staff wellness and prevent burnout included allowing staff to work remotely (57/72, 79%) and providing meals at work (45/72, 63%). If staff had a confirmed positive SARS-CoV-2 test, criteria for returning to work included a negative test (28/45, 62%) and/or being symptom-free for a site-determined duration of days (27/45, 60%).

### Testing for COVID-19

On a multiple-option question, 55/74 (74%) respondents selected that they were able to test for SARS-CoV-2 in their EDs, 23/74 (31%) selected that samples were sent to an external agency for testing, and 4/74 (5%) sites indicated that they did not perform any testing.

### Resources and Supplies

Most EDs were able to provide face masks for patients with suspected COVID-19 or influenza-like illness at arrival (70/74, 95%). Supplies in EDs that were most likely to be depleted (defined as a <14-day supply) at the time of the survey included powered air purifying respirator systems (36/58, 62%) and N-95 masks (36/69, 52%).

### Communication

Survey respondents exhibited wide variation regarding where they obtained information about the evolving pandemic and how they communicated with patients and local communities. The most common sources of information for EDs included local governments/health departments (47/70, 67%), hospital infection control practitioners (43/70, 61%), state governments (34/70, 49%), and the WHO (33/70, 47%). Common methods for communicating with the local community included social media (53/62, 85%), television (52/62, 84%), and newspapers (46/62, 74%). The most common methods for communicating updates to staff were email (58/69, 84%), website/intranet (52/69, 75%), and video conference (48/69, 70%). The most common methods for communicating updates to patients and families were flyers/posters (41/64, 64%), in-hospital TV channels/displays (37/64, 58%), and social media (34/64, 53%).

### Innovation

In an open-ended survey section, we recorded both successful and unsuccessful innovations developed in respondent EDs to address the pandemic (Table 3). Successful innovations generally included development of separate treatment spaces for COVID-19 patients and plans for local community engagement via electronic and social media. Respondents reported unsuccessful innovations related to testing capacity, PPE availability, and staffing plans.

## DISCUSSION

In this global survey, we leveraged the EMERGE network to establish rapid collaborations with non-EMERGE EDs and obtained estimates of ED pandemic preparedness and response to COVID-19 from 74 EDs in 28 countries comprising the six WHO regions. Despite substantial differences among surveyed EDs, there were many similarities in how EDs are responding to the pandemic, especially with screening protocols, capacity expansion, and staffing. Despite having disaster plans in place, globally ED pandemic preparedness and response is difficult and variable. There was a substantial human cost to the pandemic as 43% of the EDs reported providers and staff had contracted COVID-19 and missed work, further impacting the ED’s ability to provide care. Finally, EDs were willing to share protocols, lessons learned, and innovative solutions that can be rapidly disseminated via research networks such as EMERGE, or via global organizations such as the WHO to benefit emergency care globally.

Our survey identified multiple, latent patient-safety threats due to inadequate disaster preparedness. One third of respondents did not have a pandemic preparedness plan prior to the COVID-19 pandemic. These findings are consistent with another report surveying 102 pediatric EDs in Europe early in the COVID-19 pandemic, which revealed that nearly a third of EDs lacked a contingency plan for pandemics and never had simulated scenarios for such events.^15^ Second, the loss of the ED workforce, especially nursing and physician staff, either due to contracting infections with SARS-CoV-2 or required quarantine from exposure to patients with COVID-19 was very high. These results are consistent with reports from the CDC and other recent studies.^16^–^19^ Third, there was variability among EDs regarding staff backup plans. Although most EDs responded that their pre-pandemic disaster plan was a successful one, nearly half of the EDs did not have an ED backup plan for staffing during disasters, most had to create new or modify existing plans to respond to the current pandemic and reported that these plans were ineffective or only somewhat effective.

Reassuringly, most EDs recognized the impact of the pandemic on staff wellness and had developed plans for improving provider well-being. These interventions included new guidelines for remote work whenever possible, meals during shifts, childcare support, and/or additional time away from work. Disaster management experts voice the importance of frontline workers’ protection through planning, availability of PPE, and mindful staff scheduling, as well as strongly encouraging mental health and peer support with wellness initiatives. Our findings highlight the need for a more cohesive and comprehensive evaluation of existing disaster plans. Lessons learned from our survey could potentially be used to develop multidisciplinary in situ simulations to find optimal solutions for adapting to COVID-19 and other highly complex and evolving epidemics/pandemics in the future.^20^

Nearly all EDs developed site-specific protocols for screening and triage during the pandemic. These protocols included provision of masks and separating patients on arrival to the waiting room. Triage and pre-ED arrival screening involved tele-triage, phone, and video screening, the use of alternative sites (such as triage tents, triage in the car), and use of other non-ED sites (such as outpatient clinics and community centers). One site reported a separate screening facility with app-based screening prior to patient registration. Leveraging technology can reduce ED demand and enhance patient understanding of COVID-19 risks and has been used extensively for screening and contact tracing tools, such as the CDC Self-Checker and Singapore’s TraceTogether.^21^,^22^ Consistent with lessons learned from prior disasters, most EDs had developed protocols for enhancing capacity by repurposing unused or non-ED spaces and adapting them for care of COVID-19 patients. Essentially, most EDs and their hospitals had re-engineered the entire ED arrival, triage, throughput, and disposition processes with a system-wide response, postponing non-emergent surgeries and creating and/or expanding intensive care capabilities.

The ED and emergency medical services are an integral component of community-based health systems that require strengthening to respond to pandemics; however, it is imperative that we first obtain high quality, global data in a timely manner to understand the impact and subsequent responses. This was the motivation for our survey, which revealed important insights on the state of global pandemic/disaster preparedness. First, apart from regional EM organizations gathering local data, currently there is no infrastructure for assessing the state of ED systems across the globe and sharing experiences to provide rapid, reliable, and actionable data. For instance, adaptation of shared protocols and data would substantially reduce the time to implementation of interventions, underscoring the importance of ED-based research networks. Our survey was initiated in the nascent EMERGE network and we immediately recognized that despite having a “global” footprint, EMERGE is still not representative of the global ED system. It was very reassuring that we were quickly able to reach out to several non-EMERGE EDs via referral, allowing us to expand the survey participant pool from 26 EMERGE EDs in 17 countries to include an additional 51 non-EMERGE EDs from 11 countries. Second, our higher-than-average 57% response rate highlights the willingness of healthcare institutions across the world to participate and share experiences for the global benefit.^23^

Third, the diversity of comments regarding innovations in the domains of triage, testing, communication, staffing, and capacity-building provided in Table 3 can be reviewed and potentially applied immediately as the world continues to grapple with the pandemic. For instance, use of unmanned robots for telepresence, use of mobile technology for communication between patients, caregivers, and providers, or use of a dedicated website and app-based screening have been rapidly deployed. Repurposing used radiographic films as face shields to three-dimensional printing of face shields, ultraviolet lights for sterilizing PPE to conserve supplies, negative pressure single-person hoods and tents to overcome lack of negative pressure rooms, and dedicated airway teams composed of emergency, critical care and anesthesia providers are all examples of local innovations that can be applied globally. Fourth, comments on whether pandemic plans were successful or not provide insight into how EDs, institutions, communities, and others can learn. For instance, sharing protocols and processes for COVID-19, such as airway management guidelines aimed at first-pass success with use of advanced airway placement devices (video laryngoscopes), and higher doses of paralytics to reduce risk of aerosol exposure, can be implemented globally. Sharing of ED mitigation strategies early in the pandemic will enhance cross-pollination of ideas that can promote both patient and healthcare worker safety. Conversely, evidence of inconsistent communication between providers within an institution or from regulatory agencies, inadequate PPE supplies, inability to scale testing to meet demand, and wasted PPE from improper training are examples of how plans could be improved in the future.

## LIMITATIONS

Data obtained from surveys is inherently susceptible to selection and reporting bias. Furthermore, survey participation and response rates have declined over time due to limitations of the study design and inconvenience associated with completing poorly designed or frequent requests. This may be especially true during this pandemic, when multiple and often simultaneous requests for information were circulating broadly and a massive increase in scientific literature submissions related to COVID-19 were published.^24^ We mitigated some of these limitations by testing the survey for face, construct, and content validity before soliciting participation. In addition, we made it very convenient for expeditious completion by formatting most questions as closed-ended responses. We obtained a 57% response rate, which is better than most reported surveys.^23^ We also recognize that the two open-ended questions regarding effectiveness of existing disaster plans and interventions gave us rich anecdotal data on how individual EDs were responding, but we do not have details regarding their success/failure and/or impact. We plan to pursue these questions with follow-up interviews with ED leadership as our subsequent research project.

Given the extremely dynamic nature of the COVID-19 pandemic and the fact that countries were experiencing different levels of disease burden and different phases of the outbreak, the survey results represent the situation at a single point in time for the responding institutions. Thus, responses to survey questions will likely be different if we were to conduct the survey at another point in time. For instance, responses regarding availability of PPE or effectiveness of preparedness plans may differ as institutions continue to respond and adapt in real time. Ultimately, this study is hypothesis-generating and intends to demonstrate the power of our network.

## CONCLUSION

Our study cohort represents a large cross-sectional sample of ED responses to an ongoing global healthcare crisis. Despite having disaster plans in place, ED pandemic preparedness and response continue to be a challenge and there were multiple, latent safety threats to providers and patients. We believe global emergency research networks play an important role in near real-time collection of high-quality data on the epidemiology of large-scale events and can disseminate experiences and solutions that will impact healthcare outcomes for individuals, communities, and even nations.

## Supplementary Information







## Figures and Tables

**Figure:**
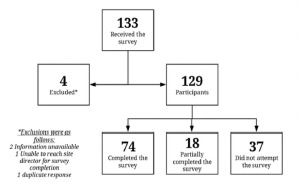
Survey responses among members of the Emergency Medicine Education & Research by Global Experts network (EMERGE) and non-EMERGE emergency departments.

**Table 1:** Strengths and weaknesses reported from the pandemic/disaster plans.

	Strengths	Weaknesses
Communication	Communication with large number of employees, interdepartmental communication, ongoing patient/physician communication, telecommunication, health care workers’ communication with government	Failure in communicating logistics
Triage	ED triage tents, pre-triage bed reassignment, creating different zones for triage based on symptoms presented	Time-consuming efforts to organize the triage plans
Testing	Creating mobile testing unit, drive-through testing units	Testing capacity was low, slow process of government approval of in-house rapid testing, difficulty in obtaining official confirmation for the need for testing, long wait time for test results, bedside equipment shortage
Supplies	Resource allocation was conducted adequately, gradually increased supplies	Difficulty in mobilization of resource, PPE shortage faced at initial stages of the pandemic, planning to secure additional PPE was a slow process
Space	Creating separate areas for patients with COVID-19 like symptoms, creating field hospitals and dedicated COVID-19 centers, creating tents, halting elective procedures, and creating space for COVID-19 patients	Creation of surge capacity for the possibility of large volume of patients with respiratory distress
Staff	Staff pooling into categories to replace staff in critical areas, smooth communication and coordination, efficient training, interdepartment training, cooperation with medical students and other health care workers, and great staff well-being initiatives	Staffing mobilization, hesitancy of certain healthcare workers, mixing staff schedule to work on all zones simultaneously, no clear direction for sick healthcare workers

*ED*, emergency department; *COVID-19*, coronavirus disease 2019; *PPE*, personal protection equipment.

**Table 2:** COVID-19 screening criteria used in participating EMERGE and non-EMERGE emergency departments.

Fever	72 (97%)
Signs/Symptoms of lower respiratory illness (cough, difficulty breathing)	71 (96%)
Close contact with a confirmed case of COVID-19	66 (89%)
Travel to affected areas	66 (89%)
Signs/Symptoms of upper respiratory illness (runny nose, sore throat)	63 (85%)
Close contact with a suspected case of COVID-19	59 (80%)
Timely relation to a possible contact (ie, 14 days)	53 (72%)
Healthcare worker	40 (54%)
Signs/Symptoms of gastrointestinal illness (vomiting, diarrhea)	36 (49%)
Nonspecific symptoms (malaise, myalgias, headache)	32 (43%)
Immunocompromised	25 (34%)

*EMERGE*, Emergency Medicine Education & Research by Global Experts; *COVID-19*, coronavirus disease 2019.

**Table 3:** Innovations reported and developed in response to COVID-19 across participating EMERGE^*^ and non-EMERGE emergency departments.

Communication	Robots for communication Tele-consultation Developed vernacular language standees Dedicated call center facility (Run by medical & nursing students & doctors) Using Zoom meeting to interact with patients regarding clinic visits, questions, etc. Conducting community awareness sessions
Triage	Teletriage system App-based screening Triage truck Designated COVID-19 center manned by ACE team Screening area with a Decon shower facility Formulated a ventilator triage protocol based on a scoring system devised from existing literature
Test	Sampling booths Door-to-door screening for all people in the community Drive-through swab for COVID-19 Results available within 90 minutes for high urgency needs and number of high urgency tests is limited to 15/day Biofire testing for patients requiring admission with results in 4–12 hours Mobile vehicle for testing
PPE	Ultraviolet sterilization of N95 masks Locally designed Intubation box Reusable ultraviolet sterilization of N95s, masks, and gowns Disposable aerosol box for airway management 3-D printer face shields and 3-D printed face masks. Visors used instead of masks Use of short-sleeved gowns instead of long sleeves due to the shortage
Area	Isolated areas, fever clinic and COVID-19 tents Field hospitals Flu-screening isolation facility for staff employed in COVID-19 ward Ambulance hall rebuilt into extra patient rooms Negative pressure room ED restructured into zones
Staff	Pre-triage screening station manned by non-medical ED staff EMCREWS team managed by a consultant (attending) working remotely Pooling of rotation forming the ACE team Hired medical students Hospital infection committee for training and certifying HCWs and allied staff in donning and doffing Fever clinics with volunteers helping in segregation and providing PPE
Other	Provide shelter options for COVID-19 positive patients Creation of SARI cubicle Closure of AC ducts Use of separate lifts/elevators

*3-D*, three dimensional; *Decon*, decontamination; *COVID-19*, coronavirus 2019; *ED*, emergency department; *SARI*, severe acute respiratory infection; *AC*, air conditioner; *Biofire*, Biofire Diagnostics: Syndromic Infectious Disease Diagnostics (Salt Lake City, UT) *ACE*, anesthesia, critical care, and emergency medicine; *HCW*, healthcare workers; *PPE*, personal protective equipment.
