# Supplementary material for: A Global Survey of Emergency Department Responses to the COVID-19 Pandemic
Source: West J Emerg Med. 2021 Aug 21;22(5):1037–44. doi: 10.5811/westjem.2021.3.50358 (PMC8463065; doi:10.5811/westjem.2021.3.50358)
Supplement: Supplementary file 1 [file wjem-22-1037-s001.docx]

*Appendix A: Survey*

**Part A: COVID-19 Response Protocols**

1. Do you have an ED protocol that is used as a guide for screening, testing, and managing patients with suspected COVID-19 infection? (yes/no/do not know)
   1. If yes – can you share it (Upload instructions)
2. When was the system wide pandemic preparedness plan activated for COVID-19?
   1. Give date if available <text/calendar>
   2. If not – give estimate
      1. November 2019
         1. First week
         2. Second Week
         3. Third week
         4. Fourth week
      2. December 2019
         1. First week
         2. Second Week
         3. Third week
         4. Fourth week
      3. January 2020
         1. First week
         2. Second Week
         3. Third week
         4. Fourth week
      4. February 2020
         1. First week
         2. Second Week
         3. Third week
         4. Fourth week
      5. March 2020
         1. First week
         2. Second Week
         3. Third week
         4. Fourth week
      6. April 2020
         1. First week
         2. Second Week
         3. Third week
         4. Fourth week
3. Why was the plan activated? <checkbox>
   1. Case identified in institution,
   2. Case identified in community,
   3. Case identified in state/province,
   4. Case identified in country,
   5. Preparation in absence of a case
4. What is your plan based on?
   1. World Health Organization (WHO)
   2. Centers for Disease Control and Prevention (CDC)
   3. Guidelines issued by your own country
   4. Guidelines developed by you own institution
   5. Others (please specify)

**Part B: – ED Triage/Screening and Capacity**

1. Triage
   1. What are your screening criteria for patients for COVID-19 that is currently being used in your ED? Select all that apply
      1. Fever
      2. Signs/Symptoms of upper respiratory illness (runny nose, sore throat)
      3. Signs/Symptoms of lower respiratory illness (cough, difficulty breathing)
      4. Signs/Symptoms of gastrointestinal illness (vomiting, diarrhea)
      5. Nonspecific symptoms (malaise, myalgias, headache)
      6. Close contact with a suspected case of COVID-19
      7. Close contact with a confirmed case of COVID-19
      8. Timely relation to a possible contact (i.e. 14 days)
      9. Other: Please specify
   2. How many times have the triage criteria changed since you have started screening for COVID-19?
      1. Not changed,
      2. Changed once,
      3. Changed twice,
      4. Changed > 2 times but less than 5 times,
      5. Changed > 5 times
   3. Do you provide face masks for patients with suspected COVID-19 or any suspected ILI (influenza like illness) at ED arrival? <drop down>
      1. Yes
         1. If yes, where/when is the mask provided
            1. At ED entrance i.e. Before triage
            2. After triage, before waiting room or ED room placement
            3. Other
      2. No
   4. Do you have separate waiting rooms/separate space in waiting room for patients with possible COVID-19 after ED triage? <drop down>
      1. Yes
         1. If yes, was this space created within existing waiting room space
      2. No
   5. What innovative triage methods have been implemented to reduce the number of patients being evaluated in the ED, select all that apply: <check box>
      1. Triage in car
      2. Tele-triage
      3. Phone screening
      4. Video triage
      5. Triage Tents
      6. Alternative sites such as outpatient clinics, community centers
      7. Other <text>
      8. None of the above
2. ED capacity
   1. Have you added additional treatment spaces to increase ED capacity? <drop down> (Yes/No)
      1. Yes
      2. No
         1. What was implemented? Select all that apply: <Checkbox>
            1. Inside the ED

Added rooms within the ED (open unused rooms)

Modified existing rooms

Added beds in hallways

Added chairs/recliners to see patients

Created space separated by curtains

- - - - 1. Outside the ED:

Existing non-ED space such as subspecialty clinics now to be used as ED rooms

Temporary rooms example – tents, mobile units, trailers

- - - - 1. Other <text>
      1. Are you **considering** any of the following to increase your ED capacity? Select all that apply: <checkbox>
         1. Inside the ED

Adding rooms within ED (open unused rooms)

Modifying existing rooms

Adding beds in hallways

Adding chairs/recliners to see patients

Creating space separated by curtains

- - - - 1. Outside the ED:

Existing non-ED space such as subspecialty clinics now to be used as ED rooms

Temporary rooms example – tents, mobile units, trailers <drop down>

- - - - 1. Other <text>

1. Hospital Capacity
   1. To improve inpatient capacity, in your estimate, has the hospital done any of the following
      1. Hospital beds
         1. Added extra hospital beds i.e. opened up spare capacity
         2. Reassigned existing beds specifically for COVID-19
      2. Postpone elective/non urgent surgical procedures
      3. Created a dedicated COVID-19 team
      4. ICU
         1. Added extra ICU beds
         2. Created a new ICU
         3. Reassigned existing beds specifically for COVID-19
      5. Other

**Part C: ED Staffing**

1. Did you have an existing back-up plan for patient surges i.e. an existing back up plan prior to COVID-19? <drop down>
   1. No
   2. Yes
      1. Can you share it? <drop down>
         1. Yes
         2. No
            1. Does it involve ED physicians and nurses and other support services? <drop down>
         3. Yes

List which ones i.e.: Select all that apply <checkbox>

physicians,

Advanced Practice providers,

trainees,

Nurses,

Others

- 1. What are the criteria for activating the existing back-up plan <text>
  2. How often have you activated that plan? <text>
  3. If unable to upload an existing plan, can you describe it

1. Did you create a new or separate ED provider back-up plan specifically for COVID-19? <drop down>
   1. No (used existing back up plan)
   2. Yes
      1. Can you share that plan?
         1. No
         2. Yes
      2. Please upload the document (as allowed) or describe the plan in the text box <document upload or free text>
      3. Does it involve non-ED personnel? <drop down>
         1. Yes
            1. List personnel involved:
         2. No
2. How is the plan activated? Select all that apply <checkbox>
   1. Page
   2. direct call,
   3. e-mail,
   4. Other <text>
3. Have you activated it for COVID-19? <drop down>
   1. Yes
   2. No
4. What is your perception about its effectiveness <Likert scale>
   1. Not effective – Somewhat effective – Very effective
   2. Any additional comments?
5. Do you have special trained COVID-19 intubation team?
   1. Yes <checkbox>
      1. Hospital inpatient team
      2. ED airway management
      3. ED intubation
   2. No
6. Do you have a state/province Comprehensive Emergency Response teams? Yes/No

**Part D: Staff wellness/burnout**

1. Has any of your staff missed work because of possible COVID-19 illness? <drop down>
   - 1. Yes
        1. Physicians (yes/no) and how many
        2. Residents (yes/no) and how many
        3. Nurses (yes/no) and how many
        4. Advanced Practice providers (nurse practitioners, physician assistants etc) (yes/no) and how many
        5. Others
     2. No
   1. Have any of your staff tested positive for COVID-19?
   2. What are the criteria/protocol for testing staff based on exposure risk and or symptoms?
   3. What are the criteria for coming back to work after exposure?
      1. Negative COVID test
      2. If COVID test positive what criteria were used to define recovery and return to work
2. What has your ED done to address staff wellness and prevent burnout?
   1. Provided meals
   2. Childcare
   3. Additional time off from work
   4. Ability to work remotely for example staff meetings conducted via web instead of in person meetings
   5. Others <open text>
3. Based on your experience, can you give suggestions on how to improve staff wellness and reduce burnout? Some of your suggestions could be useful for other institutions to learn from and possibly adopt. <open text>

**Part E: Supplies**

1. Have you run out of any personal protective equipment or PPE (gowns, goggles, masks, gloves, N95, caps)

a. If yes which ones (select all that apply)

Gowns, goggles, masks, gloves, N95, caps

1. Does the pandemic plan include obtaining/stocking the ED with additional PPE? <drop down>
   1. Yes
      1. Which kind? <open text>
      2. Has the amount of supplies included in the pandemic plan been adequate? <drop down>
         1. Yes
         2. No
   2. No

**Part F: Communication**

1. Where does your ED/institution obtain up to date information regarding COVID-19: Select all that apply <checkbox>
   1. World Health Organization
   2. CDC
   3. Hospital infection control practitioner
   4. Hospital administrator
   5. Local government / Health Department
   6. Other, please describe
2. How do you communicate with staff during the COVID-19 pandemic? Select all that apply, add frequency:
   1. Telephone Frequency
      1. Calls
      2. Texts
   2. Email Frequency
   3. Face-to-face meetings Frequency
   4. Website/intranet Frequency
   5. Social media Frequency
      1. WhatsApp,
      2. Facebook
      3. Twitter
      4. WeChat
      5. Other Frequency
3. How you are you communicating with patients/families during COVID-19 pandemic? Select all that apply <checkbox>
   1. Flyers / Posters Frequency
   2. In-hospital TV channels / Displays Frequency
   3. Telephone Frequency
   4. Email Frequency
   5. Face-to-face meetings Frequency
   6. Website/intranet Frequency
   7. Social media Frequency
   8. What’s app, or similar interface Frequency
   9. Other (please describe) Frequency
4. Are you communicating with the community at large and if so which media sources do you use to share COVID-19 pandemic information with larger groups? Select all that apply <checkbox>
   1. Newspapers
   2. TV
   3. Social media
   4. Bill boards / Posted signs
   5. Radio
   6. Other, please describe

**Part G:** **Institutional characteristics**

1. Country
2. State if applicable
3. Province
4. ED Characteristics
   1. Setting
      1. Academic / University (defined as training residents)
      2. Non-academic
      3. Private (able to refuse care)
      4. Public / Funded by Government (unable to refuse care / open to all)
      5. other
   2. Number of ED beds (fill in number)
   3. Average number of ED encounters per day (fill in number)
   4. Average number of ED encounters per year (fill in number)
   5. Distribution of visits by age
      1. Adult – (fill in % - if all adults then 100%)
      2. Pediatric - (fill in % - if all pediatrics then 100%)
      3. Adult and Pediatric - (fill in % - total adult and pediatric = 100%)
5. Hospital Characteristics (options could be empty space for number or unavailable)
   1. Number of hospital beds
   2. Number of Intensive Care Unit beds

**Part H: Preparedness**

1. Does your site routinely train for mass casualty/disaster planning? (yes/no/do not know)
   1. If yes – how often
   2. When was the last training session?
   3. How do you train
      1. Tabletop exercises
      2. Simulation drills
      3. Other (add language)
2. If your site has a pandemic plan and it was deployed
3. Do you consider the response plan successful overall?
4. What areas of the plan have been successful and why in your opinion? (could open text or provide a drop down including the sections we mentioned above: staffing, supplies, space, etc.)
5. What areas of the plan have not been successful and why in your opinion?

**Part I: Open ended** – any additional experiences or information that can be valuable as lessons learned (or learning) from COVID-19 for your site

Other sites that is applicable locally (i.e. your region/country) or internationally

**Part J**: Please suggest a reference along with contact information at an additional institution in your country that could participate in this survey:

Name:

Title:

Institution:

Contact email:

Contact telephone number:
