# Supplementary figures and images for: A Global Survey of Emergency Department Responses to the COVID-19 Pandemic
Source: West J Emerg Med. 2021 Aug 21;22(5):1037–44. doi: 10.5811/westjem.2021.3.50358 (PMC8463065; doi:10.5811/westjem.2021.3.50358)

*Appendix B: One-page infographic distributed prior to engage more participants in the survey*


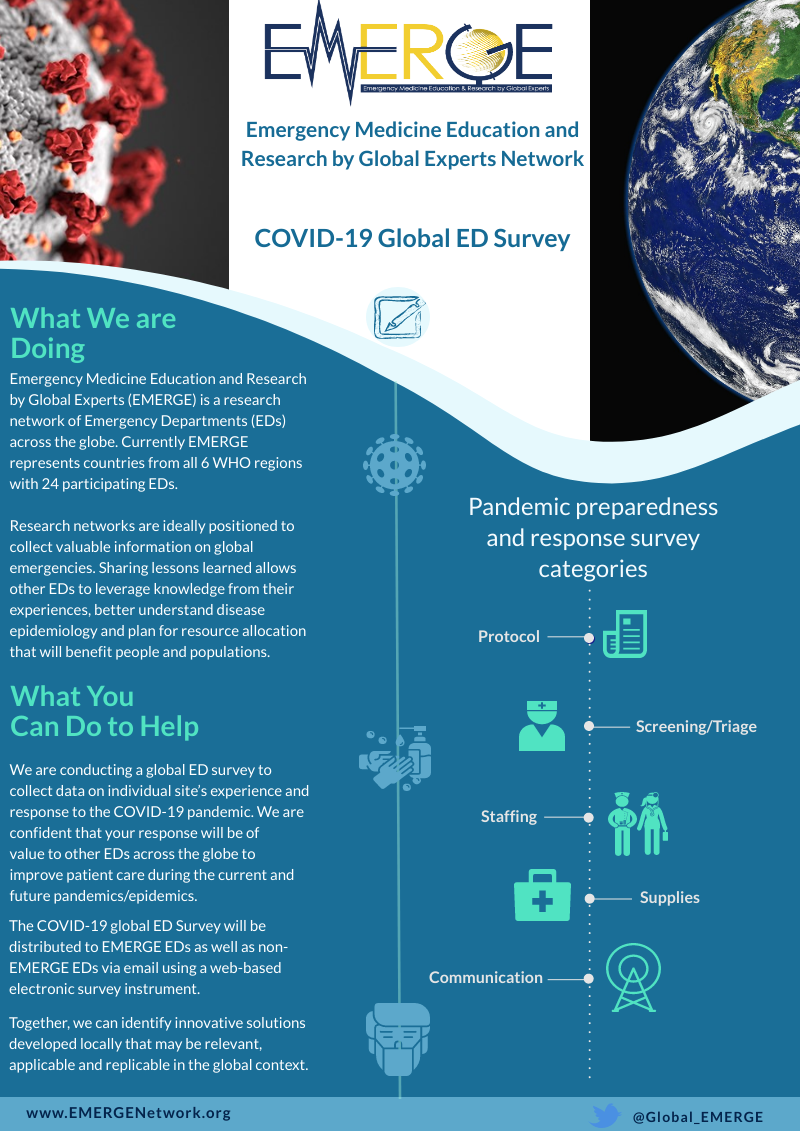

Supplement: Supplementary file 2 [file wjem-22-1037-s002.docx]
