# Supplementary material for: A Global Survey of Emergency Department Responses to the COVID-19 Pandemic
Source: West J Emerg Med. 2021 Aug 21;22(5):1037–44. doi: 10.5811/westjem.2021.3.50358 (PMC8463065; doi:10.5811/westjem.2021.3.50358)
Supplement: Supplementary file 3 [file wjem-22-1037-s003.docx]

Supplementary Table 1: Characteristics of responding EDs and prevalence of COVID-19 stratified by continents

| **Sites** | **ED Beds** | **ED Visits per day** | **Adult/Pediatric/Both** | **Academic/Non-Academic** | **Public/Private** | **COVID-19 Burden^1^** | **COVID-19 Death Toll^2^** | | **Per Capita GDP-2019 (USD)^3,4^** |
| --- | --- | --- | --- | --- | --- | --- | --- | --- | --- |
| **AFRICA** |  |  |  |  |  |  |  |  | |
| ED1 | 20 | 50 | Pediatric and Adult | Academic | Public | 1.0 | 0.1 | $1,122.12 | |
| ED2 | 100 | 70 | Pediatric and Adult | Academic |  | 60.8 | 0.6 | $2,202.12 | |
| **ASIA** |  |  |  |  |  |  |  |  | |
| ED3 | 20 | 120 | Pediatric and Adult | Academic |  | 2.3 | 0.1 | $2,099.60 | |
| ED4 | 22 | 100 | Pediatric and Adult | Academic |  | 2.3 | 0.1 | $2,099.60 | |
| ED5 | 800 | 400 | Pediatric only | Academic |  | 189.2 | 0.8 | $65,233.28 | |
| ED6 | 100 | 700 | Pediatric and Adult | Academic |  | 60.2 | 2.4 | $10,261.68 | |
| ED7 | 60 | 450 | Adult only | Academic |  | 233.5 | 1.0 | $65,233.28 | |
| ED8 | 40 | 150 | Pediatric and Adult | Academic |  | 4.6 | 0.1 | $2,099.60 | |
| ED9 | 43 | 500 | Pediatric and Adult | Academic |  | 16.4 | 0.3 | $25,941.00 | |
| ED10 | 35 | 90 | Pediatric and Adult | Academic |  | 6.5 | 0.2 | $2,099.60 | |
| ED11 | 60 | 650 | Adult only | Academic |  | 60.6 | 2.4 | $10,261.68 | |
| ED12 | 45 | 170 | Pediatric and Adult | Academic |  | 106.4 | 3.4 | $7,583.69 | |
| ED13 | 10 | 15 | Pediatric and Adult | Academic |  | 87.9 | 1.5 | $40,246.88 | |
| ED14 | 20 | 4 | Adult only | Academic |  | 90.9 | 2.2 | $40,246.88 | |
| ED15 | 8 | 90 | Pediatric only | |  | 94.3 | 2.3 | $40,246.88 | |
| ED16 | 75 | 200 | Pediatric and Adult | Academic |  | 29.8 | 2.5 | $4,135.57 | |
| ED17 | 10 | 80 | Pediatric and Adult | Academic |  | 98.0 | 2.5 | $40,246.88 | |
| ED18 | 1000 | 270 | Pediatric and Adult | Academic | Public | 2050.5 | 2.0 | $65,233.28 | |
| ED19 | 175 | 350 | Pediatric and Adult | Academic | Public | 19.1 | 0.6 | $2,099.60 | |
| ED20 |  |  | Pediatric and Adult | | | 2538.8 | 2.4 | $65,233.28 | |
| ED21 | 10 | 40 | Pediatric and Adult | Academic |  | 111.4 | 3.3 | $40,246.88 | |
| ED22 | 42 | 45 | Adult only | Academic | Public | 35.6 | 0.4 | $3,853.08 | |
| ED23 | 250 | 250 | Pediatric and Adult | Academic |  | 41.3 | 1.4 | $2,099.60 | |
| ED24 | 40 | 150 | Pediatric and Adult | Academic |  | 41.3 | 1.4 | $2,099.60 | |
| ED25 | 248 | 1200 | Pediatric and Adult | Academic |  | 8728.7 | 5.2 | $62,088.06 | |
| ED26 | 100 | 550 | Pediatric and Adult | Academic | Public | 206.1 | 3.4 | $11,414.21 | |
| ED27 | 90 | 200 | Adult only | Academic |  | 49.1 | 1.6 | $2,099.60 | |
| ED28 | 7 | 20 | Pediatric and Adult | Academic |  | 127.2 | 5.4 | $40,246.88 | |
| ED29 | 30 | 300 | Pediatric and Adult | Academic | Public | 222.4 | 3.6 | $11,414.21 | |
| ED30 |  |  | Pediatric and Adult | | | 130.4 | 8.2 | $3,485.08 | |
| ED31 | 26 | 150 | Pediatric and Adult | Academic | Public | 130.4 | 8.2 | $3,485.08 | |
| ED32 | 40 | 300 | Pediatric and Adult | | Public | 67.1 | 0.4 | $3,853.08 | |
| ED33 | 7 | 20 | Pediatric and Adult | | Private | 96.8 | 5.8 | $4,135.57 | |
| ED34 | 49 | 600 | Pediatric only | Academic |  | 61.1 | 3.4 | $10,261.68 | |
| ED35 |  |  | Pediatric only | |  | 61.1 | 3.4 | $10,261.68 | |
| ED36 | 110 | 260 | Pediatric and Adult | Academic |  | 18.2 | 0.3 | $25,941.00 | |
| ED37 | 25 | 100 | Pediatric only | Academic |  | 61.2 | 3.4 | $10,261.68 | |
| ED38 | 80 | 500 | Pediatric and Adult | Academic | Public | 61.3 | 3.4 | $10,261.68 | |
|  |  |  |  |  |  |  |  |  | |
| **AUSTRALIA** |  |  |  |  |  |  |  |  | |
| ED39 | 20 | 62 | Pediatric and Adult | Academic | Private | 242.5 | 1.5 | $55,060.33 | |
| ED40 | 36 | 250 | Adult only | Academic | Public | 249.4 | 1.7 | $55,060.33 | |
| ED41 | 47 | 300 | Pediatric and Adult | Academic |  | 291.3 | 4.0 | $55,060.33 | |
| **EUROPE** |  |  |  |  |  |  |  |  | |
| ED42 | 14 | 300 | Adult only | Academic | Public | 1316.5 | 19.2 | $50,137.66 | |
| ED43 | 166 | 830 | Adult only | Academic |  | 349.2 | 5.1 | $48,782.79 | |
| ED44 | 15 | 100 | Adult only | | Public | 1404.7 | 25.1 | $50,137.66 | |
| ED45 | 22 | 155 | Adult only | Non-Academic | Public | 1404.7 | 25.1 | $50,137.66 | |
| ED46 | 36 | 180 | Adult only | Academic | Public | 1149.0 | 103.7 | $51,615.02 | |
| ED47 | 22 | 130 | Adult only | Non-Academic | Public | 1625.9 | 43.9 | $50,137.66 | |
| ED48 | 16 | 100 | Adult only | Non-Academic | | 1583.0 | 40.4 | $46,445.25 | |
| ED49 | 80 | 180 | Pediatric and Adult | Academic | Public | 1326.8 | 140.6 | $51,615.02 | |
| ED50 | 600 | 600 | Pediatric and Adult | Academic |  | 1655.7 | 222.0 | $42,330.12 | |
| ED51 | 21 | 120 | Adult only | Academic |  | 201.4 | 20.2 | $16,731.82 | |
| **NORTH AMERICA** |  |  |  |  |  |  |  |  | |
| ED52 | 66 | 300 | Pediatric and Adult | Academic |  | 654.0 | 14.7 | $65,297.52 | |
| ED53 |  |  | Pediatric and Adult | | | 1605.9 | 62.6 | $65,297.52 | |
| ED54 | 100 | 25 | Pediatric and Adult | | Private | 1.5 | 0.2 | $1,912.90 | |
| ED55 | 100 | 215 | Pediatric and Adult | Academic |  | 1937.3 | 98.8 | $65,297.52 | |
| ED56 | 15 | 123 | Pediatric only | Academic | Public | 403.8 | 20.2 | $8,282.12 | |
| ED57 | 40 | 100 | Pediatric and Adult | Academic | Public | 22.1 | 0.6 | $4,619.99 | |
| ED58 | 65 | 232 | Pediatric and Adult | Academic |  | 2635.3 | 150.6 | $65,297.52 | |
| ED59 | 53 | 180 | Pediatric and Adult | Academic |  | 3073.8 | 176.7 | $65,297.52 | |
| ED60 | 62 | 195 | Pediatric and Adult | Academic |  | 3073.8 | 176.7 | $65,297.52 | |
| ED61 | 16 | 380 | Pediatric and Adult | Academic |  | 3073.8 | 176.7 | $65,297.52 | |
| ED62 | 38 | 175 | Pediatric and Adult | Academic | Public | 3267.1 | 191.8 | $65,297.52 | |
| ED63 | 60 | 190 | Pediatric and Adult | Academic |  | 3585.7 | 208.4 | $65,297.52 | |
| ED64 | 45 | 140 | Pediatric and Adult | Academic |  | 3653.9 | 216.0 | $65,297.52 | |
| ED65 | 150 | 300 | Pediatric and Adult | Academic |  | 3653.9 | 216.0 | $65,297.52 | |
| ED66 | 52 | 250 | Pediatric and Adult | Academic |  | 3974.8 | 240.8 | $65,297.52 | |
| ED67 | 52 | 250 | Pediatric and Adult | Academic |  | 4598.2 | 276.2 | $65,297.52 | |
| ED68 | 100 | 400 | Pediatric and Adult | Academic |  | 4802.0 | 287.8 | $65,297.52 | |
| ED69 | 25 | 100 | Pediatric and Adult | | | 4956.0 | 296.7 | $65,297.52 | |
| ED70 |  |  | Pediatric and Adult | | | 5380.8 | 317.0 | $65,297.52 | |
| ED71 | 76 | 80 | Pediatric and Adult | Academic | Public | 76.8 | 3.9 | $8,717.19 | |
| ED72 | 21 | 300 | Pediatric only | Academic | Public | 101.8 | 0.6 | $12,243.81 | |
| **SOUTH AMERICA** |  |  |  |  |  |  |  |  | |
| ED73 | 11 | 30 | Pediatric only | Academic | Public | 37.4 | 2.4 | $9,946.03 | |
| ED74 | 34 | 200 | Pediatric and Adult | Academic | Private | 423.0 | 4.6 | $14,896.45 | |
| 1. COVID-19 burden is defined as the cumulative number of COVID-19 cases as of the survey completion date, per 1,000,000 population. 2. COVID-19 death toll is defined as the cumulative number of COVID-19 deaths as of the survey completion date, per 1,000,000 population. 3. Data.worldbank.org. 2021. GDP per capita (current US$) \| Data. [online] Available at: <https://data.worldbank.org/indicator/NY.GDP.PCAP.CD> [Accessed 11 February 2021]. 4. Eng.stat.gov.tw. 2021. National Statistics, Republic of China (Taiwan). [online] Available at: <https://eng.stat.gov.tw/point.asp?index=1> [Accessed 11 February 2021]. | | | | | | | | | |
